# Case Report: Early-onset juvenile psoriatic arthritis presenting at 15 months—a diagnostic challenge in infancy

**DOI:** 10.3389/fped.2026.1779782

**Published:** 2026-05-28

**Authors:** Lana Hasasna, Mayar Atawneh, Math Atiani, Mohammad Alami, Fawzy M. Abunejma

**Affiliations:** Faculty of Medicine, Hebron University, Hebron, Palestine

**Keywords:** juvenile arthritis, juvenile idiopathic arthritis (JIA), juvenile psoriatic arthritis (JPsA), pediatric psoriasis, psoriasis

## Abstract

This case describes a rare early-onset presentation in a 15-month-old girl, who first developed guttate psoriatic lesions followed by knee arthritis confirmed by ultrasound. The patient had a strong family history of autoimmune diseases. Immunological testing, including antinuclear antibodies, was negative, but with positive non-specific inflammatory markers, such as C-reactive protein and erythrocyte sedimentation rate, offering a clue about an inflammatory process causing this presentation. The diagnosis of juvenile psoriatic arthritis was made according to the International League of Associations for Rheumatology (ILAR) Criteria for Juvenile Psoriatic Arthritis. This case highlights an uncommon presentation of juvenile psoriatic arthritis in a toddler, characterized by guttate psoriasis preceding arthritis and negative autoimmune serology. Recognition of this pattern and early imaging-confirmed synovitis supported the timely initiation of disease-modifying therapy, resulting in sustained clinical remission.

## Background

Psoriasis in children is not rare. Population estimates suggest a prevalence of approximately 2%, with approximately one-third of all psoriasis beginning in childhood (1).

The most prevalent type is chronic plaque psoriasis (psoriasis vulgaris), while guttate psoriasis accounts for nearly 15%–30% of pediatric cases. Guttate psoriasis is often preceded by streptococcal infections, although only about 10% of affected children test positive for anti-streptolysin O, and roughly 5% demonstrate positive throat cultures for Streptococcus. Typically, guttate psoriasis is self-limiting and resolves within a few months, but recurrences are common, and progression to psoriasis vulgaris may occur. Diagnosis is primarily based on clinical findings, although histopathological examination can assist in confirmation (2–6).

Psoriatic arthritis lies within the juvenile idiopathic arthritis (JIA) spectrum. It presents with chronic synovitis and carries an increased risk of asymptomatic eye inflammation, particularly uveitis (7–10).

A combination of genetic susceptibility, environmental triggers, and immune dysregulation underlie juvenile psoriatic arthritis (JPsA), which affects only 0.7%–1.2% of children with psoriasis and is therefore challenging to diagnose (6, 9).

JPsA represents approximately 5% of JIA cases. The initial presentation is commonly oligoarticular arthritis (<5 joints), although some patients may later progress to polyarticular involvement (>5 joints), particularly in the absence of treatment (7–11).

In this article, we present a rare case of a very young girl who presented with symptoms of arthritis, which was later diagnosed as psoriasis of the skin and subsequently confirmed as juvenile psoriasis of the joints.

## Case presentation

We report the case of a 15-month-old girl who presented to the pediatric clinic with a 2-month history of limping, left knee pain, swelling, and early-morning stiffness lasting more than 1 h. Subsequently, she developed swelling of small joints of both hands. This presentation was preceded by a diagnosis of psoriasis several weeks earlier.

Her family history was notable for autoimmune diseases. Her father had rheumatoid arthritis since age 13, and a cousin had Behçet's disease.

On physical examination, her growth parameters were as follows: Ht.: 76 cm (fifth percentile); Wt.: 8.9 kg (14th percentile); HC.: 46 cm (35th percentile); and body surface area: 0.4 m^2^ (fourth percentile).

Musculoskeletal system examination showed diffuse left knee swelling with limited flexion. The small joints of the hands were swollen with tenderness.

**Figure 1:**
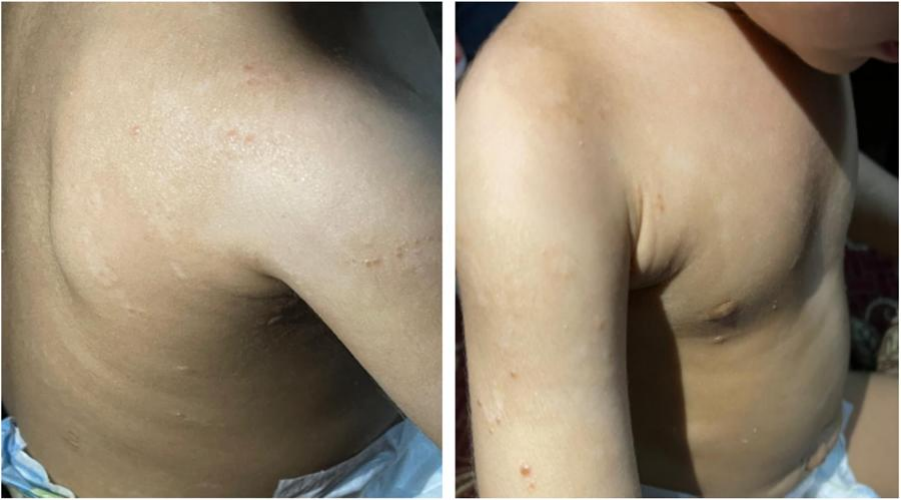
Diffuse, multiple, small, drop-like erythematous papules and plaques with silvery scales, consistent with psoriatic guttate lesions, on the trunk and the back.

On skin examination, there were diffuse, multiple, small-sized, drop-like erythematous papules and plaques, with silvery scales, consistent with psoriatic guttate lesions, distributed across the posterior side of the scalp, retro-auricular area, trunk, and back (Figure 1).

Laboratory tests revealed leukocytosis, elevated C-reactive protein (CRP: 6), and erythrocyte sedimentation rate: 60). Immunology testing revealed normal complement values (C3: 126, C4: 28) and circulating immune complexes. Antinuclear antibodies (ANAs), anti-cyclic citrullinated peptide, and rheumatoid factor (RF) were negative. The thyroid hormone profile was normal (thyroid-stimulating hormone and free thyroxine). Tissue transglutaminase immunoglobulin A and fecal calprotectin were negative.

**Figure 2:**
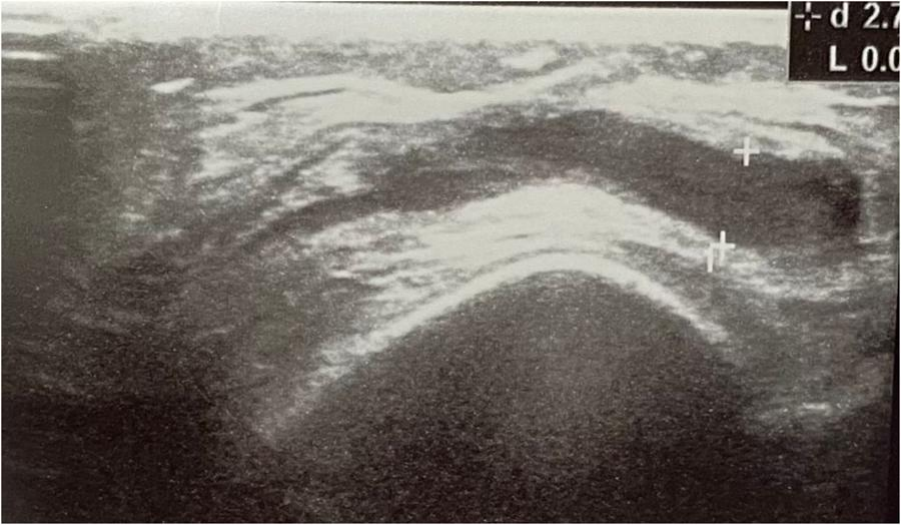
Knee ultrasound demonstrating periarticular hypoechoic synovial thickening measuring about 2.5 × 0.5 cm, suggestive of synovitis.

Ultrasound of the left knee revealed periarticular hypoechoic synovial thickening with increased surrounding vascularity on color Doppler, measuring about 2.5 × 0.5 cm, suggestive of synovitis (Figure 2). A slit-lamp exam performed by an ophthalmologist revealed no uveitis.

Reactive arthritis was considered one of the differential diagnoses; however, with no identified preceding infection and involvement of the metacarpophalangeal/proximal interphalangeal (MCP/PIP) small joints, it was effectively excluded. Inflammatory bowel disease (IBD)-associated arthropathy was also excluded given the negative fecal calprotectin and absence of gastrointestinal (GI) symptoms on thorough history.

After completing all required investigations, the diagnosis of juvenile psoriatic arthritis was considered. The plan was to start the patient on ibuprofen per os 80 mg three times a day and follow up after a week with laboratory results. However, after 1 month, the patient showed only partial improvement with persistent knee joint swelling and tenderness of the small joints of both hands. Consequently, she was started on subcutaneous methotrexate (MTX) 6 mg weekly, folic acid 5 mg weekly, and ibuprofen twice daily.

Since the age of 2 years and 1 month, the patient has been in clinical remission, characterized by resolution of joint swelling, tenderness, and psoriatic lesions. All her growth parameters improved within 4–6 months of methotrexate initiation.

## Discussion

According to the International League of Associations for Rheumatology (ILAR) Classification Criteria for Juvenile Psoriatic Arthritis, the criteria were met in this case with the presence of arthritis and psoriatic lesions (8). However, the challenging point is the development of arthritis after the onset of psoriasis, which, according to the literature, complicates only 2% of pediatric psoriasis cases (12).

The Vancouver criteria were also applied, as they are particularly helpful in pediatric cases where psoriasis may be absent at onset. In our patient, the major criteria of arthritis and psoriasis were fulfilled, while the minor criteria (dactylitis, nail pitting, family history of psoriasis, and psoriasis-like rash) were absent (13).

The rationale for presenting both frameworks is supported by Stoll et al., who found that out of 139 patients, only 59 met both criteria (ILAR and Vancouver), with 58% excluded by ILAR (14). Accordingly, we reference both ILAR and Vancouver criteria to provide a structured diagnostic justification and to acknowledge classification limitations in early/atypical pediatric presentations.

Reports describe two peaks of onset, with one peak around 2–3 years and a second in late childhood to early adolescence (about 10–12 years) (9). Zisman et al. published a study with the Rheumatology Research Alliance (CARRA) registry, reporting that the average age of symptom onset was 8.34 ± 4.57 years, with 22.7% of cases classified as early-onset (< 4 years old) (15). Our case belongs to this early-onset minority group, with an even earlier onset than the average.

A strong family history of autoimmune disease was present, which is typically associated with an early onset of the disease. Relevant family history in JPsA has been reported in 31.3%–59% of cases (15). Khani et al. reported in a case–control study that JIA patients with severe disease have a family history of autoimmune disease, unlike patients without (16). However, only one reported case of JPsA younger than 2 years exists in the literature (12), and that patient did not have a positive family history, unlike our patient, who had both a strong family history and a very early age of presentation.

This case presents with an oligoarticular onset, consistent with Butbul Aviel et al., who reported that 55% of JPsA patients had oligoarticular onset (7). In contrast, Zisman et al. reported that only 44.7% were oligoarticular (15). That study also noted that patients with a family history mostly exhibit the polyarticular subtype, which was not observed in our patient.

The type of psoriasis in this patient was guttate psoriasis, a pattern that is more often seen in children with positive family history (37.6%) and observed in 35.2% of female patients (17).

Guttate morphology is reported in approximately 30% of pediatric psoriasis cases, according to Mercy et al. Guttate lesions were found to be more frequent in severe psoriasis than in mild psoriasis (35.9% vs. 21.8%; *p* = 0.017). A prior streptococcal infection was reported by 22.1% of all patients, with about half of those cases associated with guttate psoriasis, making streptococcal infection a more common antecedent of guttate psoriasis. However, its prevalence did not differ between severe and mild disease (23.3% vs. 20.5%; *p* = 0.683) (18).

In our case, the patient presented with psoriatic lesions on the scalp, retroauricular areas, and trunk. In a cross-sectional study of 416 children, scalp involvement was the most common pattern (16.8%) and often co-occurred with retroauricular lesions, and nail psoriasis (1.7%), which is considered uncommon in this age group in comparison with adults, was one of the most common features of psoriasis (19–21).

Chronic anterior uveitis is reported in roughly 20% of early-onset oligoarticular juvenile rheumatoid arthritis cases, associated with ANA positivity. Our patient had negative ANA and, despite an early-onset oligoarticular course, did not develop uveitis (22–24).

Our patient's CRP levels were elevated. Notably, CRP may be normal in many patients with psoriatic arthritis (PsA). Sokolova et al., in a cross-sectional study, about *circulating biomarkers and disease activity in psoriatic arthritis,* found elevated levels in only 33% of 15 adult PsA patients (25), and Butbul Aviel et al. reported in a cohort study, only a small average rise (0.6 ± 1) mg/dL (7).

According to the literature, the early-onset presentation is more frequently linked with a positive ANA test, with 46% of cases reporting positive ANA results, emphasizing the association between the age of onset and the positivity of these antibodies (15, 22). A cross-sectional analysis further demonstrated that 61.8% early-onset patients had positive ANA tests compared with 41.4% positivity in the late-onset group (15). However, in our case, ANA testing reported negative results despite the early onset of presentation. It was reported that the oligoarticular ANA-negative arthritis is more typical in adults (22). On the other hand, the negativity of the RF finding is consistent with the literature, where the rate of positive RF is low (3%–4.7%) (7, 15, 22).

The absence of ANA positivity, uveitis, or pronounced dactylitis in our early-onset patient is noteworthy. A prior study encompassing 87 patients with juvenile psoriatic arthritis, including 12 under the age of five, categorized as the early-onset group, identified a higher prevalence of dactylitis, uveitis, and ANA positivity relative to the late-onset group. The discrepancies observed in our case may be attributed to the exceptionally early age of onset, which may preclude the manifestation of typical clinical features. Notably, patients within the early-onset group in that study had a mean age of 2.8 years (standard deviation = 1.2), with all individuals diagnosed at an age older than our patient. This indicates that the unique characteristics of our patient's condition may not align with the findings described in the literature (26).

Non-steroidal anti-inflammatory drugs and glucocorticoids—administered either orally or via intra-articular injection (23, 26)—are given as first-line treatment to alleviate symptoms and serve as a bridge treatment until long-term control is achieved. However, our patient's symptoms did not improve while using them.

Disease-modifying antirheumatic drugs (DMARDs) are the cornerstone of second-line management in children with polyarticular involvement (27). We started our patient on MTX, which is the most widely used DMARD (28). MTX is generally well tolerated in pediatric patients, although adult studies have demonstrated a relatively higher incidence of hepatotoxicity in PsA compared to rheumatoid arthritis (28, 29).

## Conclusion

This case is notable not only for the rarity of JPsA but also for the very early age of onset. Such presentations should raise more awareness, prompt thorough investigations, and encourage further research. Early recognition allows timely initiation of disease-modifying therapy and monitoring for ocular involvement or other complications, resulting in improved prognosis.

## Data Availability

The original contributions presented in the study are included in the article/Supplementary Material, further inquiries can be directed to the corresponding author.
